# Leaky lysosomes in lung transplant macrophages: azithromycin prevents oxidative damage

**DOI:** 10.1186/1465-9921-13-83

**Published:** 2012-09-24

**Authors:** H L Persson, Linda K Vainikka, Maria Sege, Urban Wennerström, Sören Dam-Larsen, Jenny Persson

**Affiliations:** 1 Division of Pulmonary Medicine, Department of Medical and Health Sciences, Faculty of Health Sciences, Linköping University, Linköping, Sweden; 2 Department of Respiratory Medicine UHL, Centre of Surgery and Oncology, County Council of Östergötland, Linköping, SE-581 85, Sweden; 3 Division of Experimental Pathology, Department of Clinical and Experimental Medicine, Faculty of Health Sciences, Linköping University, Linköping, SE-581 85, Sweden; 4 Division of Pulmonary Medicine, Department of Medical and Health Sciences, Faculty of Health Sciences, Linköping University, Linköping, Sweden; 5 Department of Respiratory Medicine UHL, Centre of Surgery and Oncology, County Council of Östergötland, Linköping, SE-581 85, Sweden; 6 Division of Medicine, Hospital of Västervik, Västervik, SE-593 81, Sweden; 7 Division of Medicine, Hospital of Eksjö, Eksjö, SE-575 81, Sweden; 8 Division of Pulmonary Medicine, Ryhov Hospital, Jönköping, SE-551 85, Sweden

**Keywords:** Apoptosis, Bronchiolitis, Ferritin, Fibrosis, Inflammation, Iron, Macrophage

## Abstract

**Background:**

Lung allografts contain large amounts of iron (Fe), which inside lung macrophages may promote oxidative lysosomal membrane permeabilization (LMP), cell death and inflammation. The macrolide antibiotic azithromycin (AZM) accumulates 1000-fold inside the acidic lysosomes and may interfere with the lysosomal pool of Fe.

**Objective:**

Oxidative lysosomal leakage was assessed in lung macrophages from lung transplant recipients without or with AZM treatment and from healthy subjects. The efficiency of AZM to protect lysosomes and cells against oxidants was further assessed employing murine J774 macrophages.

**Methods:**

Macrophages harvested from 8 transplant recipients (5 without and 3 with ongoing AZM treatment) and 7 healthy subjects, and J774 cells pre-treated with AZM, a high-molecular-weight derivative of the Fe chelator desferrioxamine or ammonium chloride were oxidatively stressed. LMP, cell death, Fe, reduced glutathione (GSH) and H-ferritin were assessed.

**Results:**

Oxidant challenged macrophages from transplants recipients without AZM exhibited significantly more LMP and cell death than macrophages from healthy subjects. Those macrophages contained significantly more Fe, while GSH and H-ferritin did not differ significantly. Although macrophages from transplant recipients treated with AZM contained both significantly more Fe and less GSH, which would sensitize cells to oxidants, these macrophages resisted oxidant challenge well. The preventive effect of AZM on oxidative LMP and J774 cell death was 60 to 300 times greater than the other drugs tested.

**Conclusions:**

AZM makes lung transplant macrophages and their lysososomes more resistant to oxidant challenge. Possibly, prevention of obliterative bronchiolitis in lung transplants by AZM is partly due to this action.

## Background

Lung macrophages function as large recipients of iron (Fe) [1-5]. By depositing ferruginous material inside their lysosomes potentially hazardous Fe becomes separated from reactive oxygen species (ROS) by intracellular antioxidative enzyme systems [1-5]. Following the enzymatic digestion of degradable Fe-containing material (executed by lysosomal enzymes working at acidic pH), liberated Fe becomes re-utilized in different cellular processes that require this metal [6,7]. However, lysosomes are acidic (pH 4.5-5.5) and rich in reducing equivalents (*e.g.,* cysteine); thus, free or loosely bound lysosomal Fe will partly exist in a redox-active ferrous state (Fe^2+^) [8,9]. If hydrogen peroxide (H_2_O_2_) escapes the protective shield of antioxidants, aggressive hydroxyl radicals (HO^·^) or similarly reactive Fe-centered radicals may be generated inside lysosomes by Fenton-type chemistry (Fe^2+^ + H_2_O_2_ → Fe^3+^ + HO^-^ + HO^·^) [3-6,10-12]. The ensuing oxidative damage on the lysosomal membranes, which leads to lysosomal membrane permeabilization (LMP) and the leakage of lysosomal Fe and hydrolytic enzymes in to the cytosol, may result in cell death [3-6,10-12]. The cytosolic enzymes caspase-3 and −9, which are regarded as key mediators of apoptosis, may then become activated [13,14]. If the cell death is extensive, the lung macrophages often fail to phagocytose all of the apoptotic cells, and the resulting post-apoptotic necrosis may promote inflammation and fibrosis [10-12,15-19].

The bronchiolitis obliterans syndrome (BOS) is a fibro-proliferative disease of poorly understood etiology that is characterized by an irreversible decline in allograft function due to fibrotic remodeling of small airways, *i.e.* obliterative bronchiolitis (OB) [20]. The macrolide antibiotic azithromycin (AZM) is a promising drug for the prevention of (BOS) [21]. Recently, a randomized, double-blind, placebo-controlled study provided evidence that lung allograft recipients, who received a low-dose of AZM (250 mg three times per week) continuously from the time of the post-transplantation hospital discharge, demonstrate a significantly lower incidence of BOS over a 2-yr follow-up period (12.5% compared to 44.2% in those who received placebo) [21]. Previous observations on chronic inflammatory lung disease support the idea that the protective effect of AZM on the airways is anti-inflammatory/immunomodulatory rather than antimicrobial [22,23]. AZM enters cells and lysosomes by nonionic diffusion [24-26]. The molecule is amphiphilic bearing two basic functions with appropriately weak pKa values [8.1 for the endocyclic tertiary amine and 8.8 for the tertiary amine carried by one of the two sugar moieties (desosamine)] [24-26]. Thus, AZM is a weak base and lysosomotropic, *i.e.,* AZM is protonated, trapped and concentrated up to > 1000-fold inside the acidic lysosomes [24-26].

Previously, we have shown that weak bases may attenuate the reactivity of lysosomal Fe, which protects lysosomes and cells against oxidative challenge [27-29]. This effect is achieved by the drug either *(i)* similar to the radio-protective agent amifostine and the synthesized derivative of the antioxidant α-lipoamide, α-lipoic acid-PLUS, which work as Fe-chelators [27,28] by stably binding intra-lysosomal Fe, or *(ii)* by raising the pH in the acidic vacuome, which blocks the uptake of Fe from the transferrin/transferrin-receptor complex in late endosomes and/or inhibits the enzymatic liberation of Fe from Fe-rich organic elements such as ferritin and worn-out mitochondria inside lysosomes [29].

Building on this previous research, we tested and found for the first time that the lung macrophages (and their lysosomes) from lung transplant recipients without AZM treatment are more susceptible to an oxidant challenge than the lung macrophages that originate from healthy subjects. In contrast, oxidant-exposed macrophages from transplant recipients treated with AZM did not exhibit lysosomal damage and dead cells were few. Results from oxidant-challenged murine macrophage-like J774 cells indicate that the cytoprotective potency of AZM on a molar basis is much greater than that of a high-molecular-weight derivative of the Fe chelator desferrioxamine (H-DFO) or ammonium chloride (NH_4_Cl).

## Methods

### Ethical considerations

The study protocol was approved by the local Ethical Committee (Linköping, Sweden) according to the guidelines of the Helsinki Declaration.

### Study population

Following informed consent, bronchoscopies with bronchoalveolar lavage (BAL) were carried out. The control group was 7 healthy, non-smoking subjects [in all cases pulmonary disease was thoroughly ruled out by a chest X-ray, lung function test and bronchoscopy]. The lung transplant recipients were 5 patients without AZM treatment who underwent surveillance post-transplantation BAL with transbronchial lung biopsies at 3 and 6 months and 3 patients with AZM treatment (250 mg three times per week) who were investigated to rule out rejection and/or infection. Transplant recipients with AZM were transplanted 11, 6 and 2 yrs before bronchoscopy and were treated with AZM for 42, 1 and 2 months, respectively. All transplant recipients received conventional triple-drug immunosuppression [with methylprednisolone, a calcineurin inhibitor (cyclosporine A or tacrolimus) and a cytostatic agent (mycophenolate mofetil)], conventional infectious prophylaxis [for cytomegalovirus, *Aspergillus* spp. and *Pneumocystis* spp.] and conventional prophylaxis for gastro-esophageal reflux (with the proton pump inhibitor omeprazol). None of the transplant recipients were diagnosed with infection and/or rejection when the bronchoscopy was performed. The demographic details of the subjects and the BALF characteristics are presented in Table 1.

**Table 1:** Patient and BALF characteristics of the population studied

	**Lung transplant recipients without AZM (n = 5)**	**Lung transplant recipients with AZM (n = 3)**	**Healthy control patients (n = 7)**
**Mean age**	48 ± 18	44 ± 13	60 ± 13
**Gender**	4 females/1 male	2 females/1 male	2 females/5 males
**BAL recovery (ml)**	122 ± 20	100 ± 8	110 ± 18
**Cell count**			
**(10**^**6**^**cells/L)**	308 ± 139	268 ± 84	244 ± 184
**% macrophages**	84 ± 14	80 ± 9	92 ± 5
**% lymphocytes**	13 ± 10	6 ± 4	4 ± 3
**% neutrophils**	3 ± 6	14 ± 10	4 ± 4
**% eosinophils**	0 ± 0	0 ± 0	0 ± 1
**% basophils**	0 ± 0	0 ± 0	0 ± 0

Data are presented as the means ± 1 SD. *BAL* bronchoalveolar lavage.

### Cell cultures and treatments

Briefly, BAL was performed by a standardized washing of the middle lobe or lingula of the lung (transplant) for 6–9 times with 20 mL of sterile 0.9% (w/v) saline solution during a fibreoptic bronchoscopy [12]. None of the BAL fluid (BALF) was stained with blood. The BALF samples were centrifuged at 200xg for 10 min. Leucocytes from the BALF were counted and seeded in 35 mm Petri dishes (± cover slips) and the lung macrophages were allowed to attach. Concomitantly, murine J774 macrophages were seeded at the same density. After a thorough rinse in phosphate-buffered saline (PBS), the dishes, which contained approximately 0.4 x 10^6^ attached lung macrophages or J774 cells/dish, were returned to standard culture conditions. Both cell types were cultured in Dulbecco's Modified Eagle’s Medium supplemented with 100 IU/ml penicillin, 100 μg/ml streptomycin, 0.25 μg/ml amphotericin B (all from GIBCO, Paisley, UK) and 10% fetal bovine serum (PAA Laboratories GmbH, Pasching, Austria) under standard culture conditions for 48 h. Before the experiments, all dishes were thoroughly rinsed in PBS. J774 macrophages were pre-treated (or not) as described below and then rinsed again. Both cell types were oxidatively stressed by glucose oxidase (GO; Sigma-Aldrich Inc., St. Louis, MO, USA). GO was added directly to the culture medium for 60 min under standard culture conditions. Under these conditions, a stable concentration of approximately 120 μM H_2_O_2_ was generated in the culture medium.

By employing J774 macrophages, the protection afforded by AZM on oxidant-challenged lysosomes and cells were further explored. For this purpose we used two reference compounds, both well known to attenuate the reactivity of lysosomal Fe. Thus, the pre-treatments were with AZM at 33–660 μM for 45 min to 4 hrs, H-DFO (which is a strong Fe chelator that exclusively targets the lysosomal compartment) at 2 mM for 4 hrs [30], or NH_4_Cl at 10 mM for 4 hrs, which blocked the enzymatic liberation of Fe in lysosomes by raising the pH to reduce the amount of available lysosomal Fe [29]. Ampoules of AZM (Pfizer Inc., Sweden) for intravenous use were reconstituted with distilled water to 100 mg/mL and diluted to the required concentrations with distilled water. The appropriate concentrations and exposure time that were utilized for NH_4_Cl and H-DFO were based on previous studies on J774 macrophages, which demonstrated optimal reduction of lysosomal Fe reactivity and maintained viability [29,30].

To confirm the lysosomotropism of AZM, J774 macrophages were pretreated with 10 mM NH_4_Cl for 5 min (which raised the lysosomal pH and largely prevented the lysosomal uptake of AZM) and then exposed to AZM in the continued presence of NH_4_Cl. Following its dissociation to NH_3_, H^+^ and Cl^-^, NH_3_ (pKa = 9.2) becomes protonated and trapped in the lysosome as NH_4_^+^, which substantially increases the intralysosomal pH [31] and prevents the accumulation of lysosomotropic substances with lower pKa values, such as AZM [27].

### Prussian blue staining of ferric Fe

The cells grown on cover slips were fixed in paraformaldehyde and stained for ferric Fe (Fe^3+^) by the Prussian blue staining procedure [11]. The cellular content of Fe^3+^ was scored independently by two examiners according to the method reported by Golde *et al.*[32]. Thus, 200 lung macrophages/cover slip were counted, and each macrophage was graded on a scale of 0–4; 0 = no blue color, 1 = faint blue staining in cytoplasm, 2 = dense blue color in minor portion of cytoplasm or medium color intensity throughout cell, 3 = deep blue staining in most of cytoplasm, 4 = dark blue throughout cytoplasm. A mean score, *i.e.* the Golde Index, for 100 macrophages was then calculated; zero being the minimum and 400 the maximum score.

### Determination of total cellular Fe, H-ferritin and reduced glutathione

The total cellular Fe was estimated using atomic absorption spectrophotometry, equipped with an iron lamp (243.3 nm), with a lower detection limit of 0.65 μg Fe/L [11]. The cellular expression of the ferritin heavy (H-ferritin) chain in cell lysates was determined by an ELISA analysis (MyBioSource, CA, USA) according to the manufacturer's instructions. Reduced glutathione (GSH) was estimated by the Glutathione Assay Kit (BioCat GmbH, Heidelberg, Germany) using a Wallace 1420 Victor Plate Reader (PerkinElmer, Waltham, MA, USA). The Fe, H-ferritin and GSH contents were normalized to the protein concentration of each sample [11].

### Assessment of lysosomal membrane permeabilization

Acridine orange (AO; Gurr, Poole, Dorset, UK), which is a metachromatic fluorophore and lysosomotropic weak base (pKa of ~ 10), is retained in its charged form (AOH^+^) inside acidic lysosomes. Using flow cytometry, the lysosomal leakage of AO (and protons) in to the cytosol of lung macrophages and J774 cells was assessed by determination of the mean value of AO-green fluorescence (the AO-relocation test) [27]. Upon blue light excitation, the AO that is bound to proteins and DNA in the cytosol and nuclei emits a weak green fluorescence, which increases when the lysosomes leak AO. Alternatively, the AO is highly concentrated in lysosomes and emits an intense red fluorescence upon excitation. The AO-relocation test is sensitive and able to monitor early and minor LMP. The method was thoroughly standardized for lung macrophages using identical settings and J774 macrophages, equally exposed to oxidants in parallel experiments, were used as a reference. Briefly, the cells were rinsed in PBS, stained with AO (2.5 μg/mL) for 15 min at 37°C, rinsed 3 times with complete culture medium, oxidatively stressed in the presence of GO and rinsed with culture medium before the analysis was performed immediately after the 1-h oxidant exposure. To initiate controlled LMP the synthetic lysosomotropic detergent *O*-methyl-serine dodecylamide hydrochloride (MSDH; kindly provided by Gene M. Dubowchik, Bristol-Myers Squibb, Wallingford, CT, USA) was used. The green (FL1 = 530 nm) fluorescence was recorded on log scale using a BD LSR Flow Cytometer (Becton-Dickinson, Mountain View, CA, USA) that was equipped with a 488-nm exciting argon laser. CellQuest software was used for data acquisition and analyses. Data are expressed as arbitrary units (A.U.).

Micrographs of J774 macrophages exposed to MSDH were also taken. Briefly, cells seeded on cover-slips were incubated with AO (2.5 μg/mL) for 15 min at 37°C, washed with PBS, and placed on the stand of a LMS Zeiss laser scanning confocal microscope. AO was excited using a 488 nm light from a 100-mW diode laser, and leakage of AO (and concomitant loss of the lysosomal proton gradient) during the MSDH exposure was followed by laser scanning micrographs in a channel defined by band-pass filters for 495–555 nm and 630 nm.

### Cell death assays

The frequency of cells with an apoptotic or necrotic morphology (*i.e.,* cytoplasmic budding/pycnotic fragmented nuclei/apoptotic bodies and membranous rupture/nuclear swelling, respectively) was estimated by phase contrast microscopy in a blinded fashion. 0.5 × 10^3^ cells/dish were counted in preselected fields. Ten hrs after ended oxidative stress, the fractions of cells (apoptotic or necrotic)/initial numbers of cells within the fields were determined. Detached cells were all necrotic. At the same time point the fraction of fragmented apoptotic DNA was assessed using hypotonic propidium iodide (Sigma Chemical Co., St. Louis, MO, USA) and cytofluorimetric analysis [11]. According to the manufacturer’s instructions, the fluorescence of AMC (7-amino-4-methyl-coumarin), which was liberated from Ac-DEVD-AMC (Becton-Dickinson, Mountain View, CA, USA) by active caspase-3-like caspases, was also analyzed in lung macrophages using a Wallace 1420 Victor Plate Reader (PerkinElmer, Waltham, MA, USA) during the 1-h oxidant challenge and up to 5 h after the end of the oxidative stress. The increase of caspase-3 as a percent of the control was determined, and the peak value was recorded.

#### Statistical analysis

The results were reported as the means ± 1 SD. Statistical comparisons were made using an ANOVA followed by a Tukey’s post-hoc test for p < 0.05 (*), p < 0.01 (**) and p < 0.001 (***).

## Results

### Lung transplant macrophages are more susceptible to oxidants if not treated with AZM

**Figure 1:**
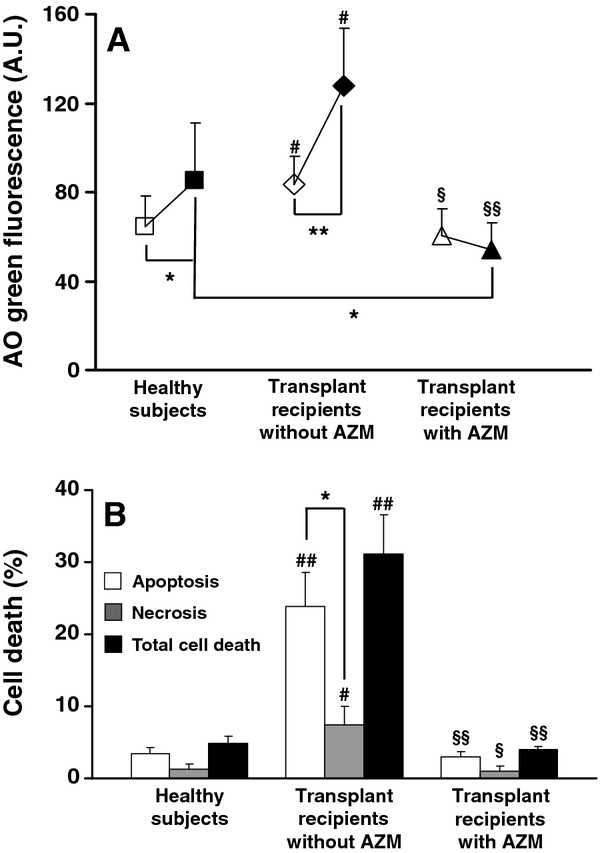
**(A) Lysosomal membrane permeabilization (LMP) and (B) cell death (apoptosis, necrosis and total cell death) in cultures of oxidatively stressed human lung macrophages that were retrieved from healthy subjects (n = 7), lung transplant recipients without azithromycin (n = 5) and with azithromycin treatment (AZM; 250 mg three times per week) (n = 3).** LMP was assessed by cytosolic/nuclear green fluorescence due to acridine orange leakage into the cytosol (arbitrary units; A.U.) in non-oxidatively stressed lung macrophages (open symbols) and oxidatively stressed lung macrophages immediately after a 1-h oxidant exposure (filled symbols). Cell death, which appeared 10 h after the end of oxidant challenge, was assessed by typical morphology using a phase contrast microscope and by the fraction of apoptotic DNA (both methods gave similar results for apoptosis). In the non-oxidatively stressed cultures, cell death was < 3%. For details, see the Methods section. Values are the means ± 1 SD. Significant differences are indicated as follows: * p < 0.05 and ** p < 0.01, ^#^ p < 0.05 and ^##^ p < 0.01 (*vs.* healthy subjects), ^§^ p < 0.05 and ^§§^ p < 0.01 (*vs.* transplant recipients without AZM).

The baseline green AO fluorescence values of lung macrophages from lung transplant recipients without AZM were significantly higher than those of the lung macrophages from healthy subjects. This observation might indicate that lysosomes in the lung transplant macrophages were more prone to leakage from the start and/or that the content of AO-binding proteins and DNA was higher than in the macrophages from healthy subjects (Figure 1A). However, following oxidative stress, macrophages from transplant recipients without AZM exhibited significantly higher green AO fluorescence values that corresponded to more lysosomal leakage of AO compared to the macrophages from healthy subjects (Figure 1A). Indeed, upon an oxidant challenge, the increase of green fluorescence was 44 ± 18 A.U. in the transplant macrophages, but only 19 ± 16 A.U. in the lung macrophages from healthy subjects (p < 0.05). In contrast, the macrophages from lung transplant recipients with AZM displayed a slight decrease of AO green fluorescence (−6 ± 6) upon oxidant challenge, which was significantly less than the increase of AO green fluorescence observed in the macrophages from the healthy subjects (p < 0.01) (Figure 1A).

Compared to cultures of lung macrophages from the healthy subjects, cultures of macrophages from transplant recipients without AZM displayed significantly more dead cells, including both apoptotic (p < 0.01) and necrotic cells (p < 0.05) (Figure 1B). In cultures of macrophages from transplant recipients with AZM apoptosis and necrosis were significantly less than in cultures of macrophages from transplant recipients without AZM (p < 0.01 and p < 0.05, respectively) (Figure 1B).

Apoptosis is often associated with increased activity of caspase-3. Correspondingly, oxidant-challenged macrophages from transplant recipients without AZM expressed high peak values of caspase-3 activation (321;% of controls). However, apoptosis may also occur without caspase-3 activation [33]. This possibility and significant necrosis might explain the great variation found (1 SD = 571%). Consequently, the caspase-3 activation observed in oxidatively stressed macrophages from recipients without AZM this did not statistically differ from the caspase-3 activation observed in macrophages from healthy control subjects (45 ± 54; mean value ± 1 SD;% of controls). This analysis was not performed on macrophages from transplant recipients with AZM because of limited sample size.

### Iron is increased in lung transplant macrophages

**Figure 2:**
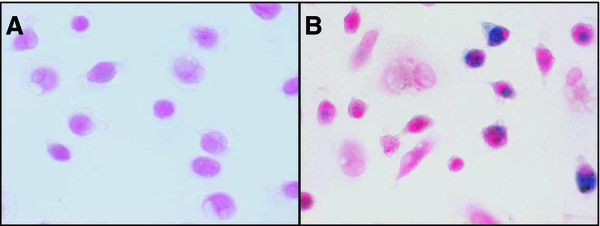
**Prussian blue-stained Fe in lung macrophages that were harvested from (A) a healthy subject and (B) a lung transplant recipient (without azithromycin).** Note the greater amount of Fe in the lung macrophages from the lung transplant recipient and a striking heterogeneity in the distribution of Fe.

Cell features that are decisive for lysosomal Fe reactivity were evaluated in the lung macrophages and the results are summarized in Table 2. The mean value for the Golde index, which is based on a cytochemical staining of ferric Fe, of macrophages that were retrieved from transplants recipients without and with AZM were significantly increased compared to that of the healthy control subjects (55.8 ± 37.2 (p < 0.05), 71.7 ± 15.1 (p < 0.05) and 8.2 ± 11.9, respectively). The Golde index normally ranges from 4 to 25 [34]. Figure 2 illustrates representative lung macrophages from healthy subjects and lung transplant recipients. Total cellular Fe assessed by atomic absorption demonstrated significantly more Fe in macrophages from transplant recipients without AZM (p < 0.05) and with AZM (p < 0.05) than in macrophages from healthy subjects. Indeed, there was even significantly more Fe in macrophages from transplant recipients with AZM than in macrophages from transplant recipients without AZM (p < 0.05). The cellular levels of the major antioxidant GSH in the macrophages from transplant recipients without AZM and healthy subjects were similar, while GSH was significantly decreased in macrophages from transplant recipients with AZM (p < 0.05; compared to the macrophages from healthy subjects). Compared to healthy subjects, the level of the cytoprotective H-ferritin in the macrophages from transplant recipients without AZM was slightly but not significantly increased. This assay was not performed on macrophages from transplant recipients with AZM due to limited sample size.

**Table 2:** **Cell features that are decisive for lysosomal Fe**^**2+**^**-reactivity in human lung macrophages**

***ASSAYS***	**Lung transplant recipients without AZM (n = 5)**	**Lung transplant recipients with AZM (n = 3)**	**Healthy control patients (n = 7)**
**Golde Index**	55.8 ± 37.2 (0.024)	71.7 ± 15.1 (0.013)	8.2 ± 11.9
**Cellular iron (ng/mg protein)**	11.2 ± 7.8 (0.048)	29.6 ± 23.6 (0.028)	4.5 ± 2.5
**H-ferritin (ng/mg protein)**	94.2 ± 76.3 (0.136)	*Not done	29.8 ± 19.8
**GSH (ng/mg protein)**	40.8 ± 20.1 (0.890)	28.3 ± 3.9 (0.012)	39.9 ± 8.1

Data are presented as the means ± 1 SD. P-value *vs.* healthy control patients is indicated. *The assay was not performed because of the size of the sample.

### Azithromycin efficiently protects J774 macrophages and lysosomes against oxidants

**Figure 3:**
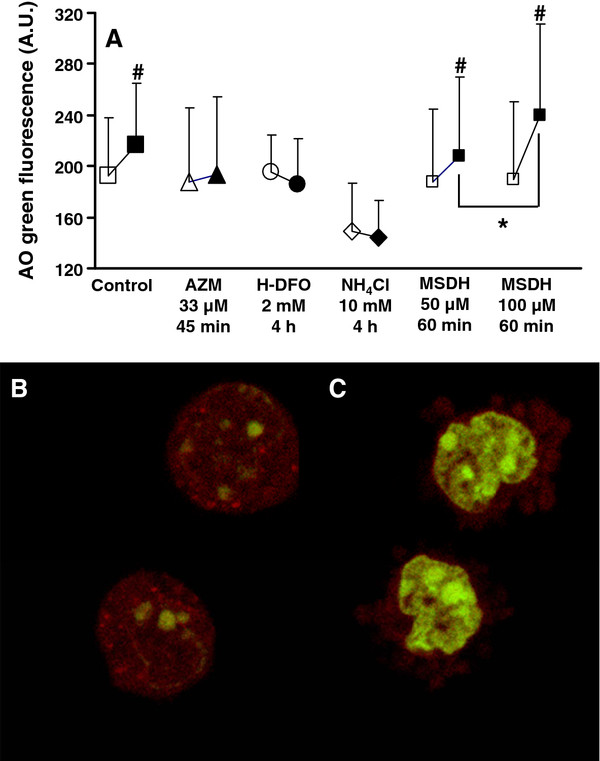
**(A) Lysosomal membrane permeabilization (LMP) in cultures of murine J774 macrophages that were treated with azithromycin (AZM) for 45 min or high-molecular-weight desferrioxamine (H-DFO)/ammonium chloride (NH**_**4**_**Cl) for 4 h at the indicated concentrations and then oxidatively stressed.** To initiate controlled LMP the lysosomotropic detergent MSDH was used. LMP was assessed by the cytosolic/nuclear green fluorescence due to the acridine orange leakage into the cytosol (arbitrary units; A.U.) in non-oxidatively stressed/non-MSDH treated cells (open symbols) and in oxidatively stressed/MSDH treated cells immediately after a 1-h oxidant exposure (filled symbols). NH_4_Cl-treated J774 macrophages exhibited pronounced granularity (observed by phase contrast microscopy) that resulted in a reduced emission of green fluorescence. Values are the means ± 1 SD (n = 6; independently performed). Significant differences are indicated as ^#^ p < 0.05 (cells either oxidatively stressed or MSDH treated *vs.* non-oxidatively stressed/non-MSDH treated control cells). * p < 0.05. (**B**) Detailed micrograph of control cells stained with AO. Lysosomes loaded with AO appear as red-fluorescent dots, while AO at a low concentration (*e.g.* AO-binding nuclear structures) emits a weak green fluorescence. (**C**) Detailed micrograph of the same cells following treatment with 200 μM MSDH for 5 min. Note cell shrinkage, an almost complete loss of intact red-fluorescent lysosomes and a massive leakage of AO into the cytosol, the latter resulting in a strong green fluorescence emitted from the nucleus and the surrounding cytoplasm.

A 4-h exposure to a range of AZM concentrations (33–660 μM) effectively protected lysosomes in J774 macrophages against an oxidant challenge (data not presented). As opposed to 4-h exposures to millimolar concentrations of H-DFO or NH_4_Cl, even a short exposure time (45 min) to a low concentration (33 μM) of AZM proved to be equally efficient (Figure 3A). Importantly, oxidant-induced LMP was prevented by micromolar concentrations of AZM, while millimolar doses of H-DFO or NH_4_Cl for 4 h were needed to achieve full protection of the lysosomes against oxidant injury (Figure 3A).

In control experiments, we observed that a 1-h exposure to 50 and 100 μM of the lysosomotropic detergent MSDH resulted in a significant increase of AO-green fluorescence (p < 0.05 and p < 0.05, respectively), which was dose-dependent (p < 0.05) (Figure 3A). Figure 3B and 3C are detailed micrographs of J774 macrophages before (Figure 3B) and after exposure to 200 μM MSDH (Figure 3C). Within a few minutes MSDH caused extensive lysosomal disruption resulting in a massive leakage of AO into the cytosol (Figure 3C).

**Figure 4:**
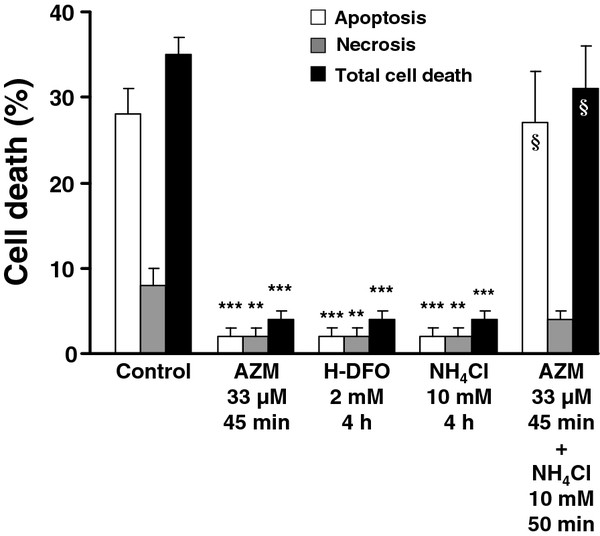
**Cell death in cultures of murine J774 macrophages that were treated with azithromycin (AZM) for 45 min or high-molecular-weight desferrioxamine (H-DFO)/ammonium chloride (NH**_**4**_**Cl) for 4 h at the indicated concentrations and then oxidatively stressed.** Cell death, which appeared 10 h after the end of the oxidant challenge, was assessed by typical morphology using a phase contrast microscope and by the fraction of apoptotic DNA (both methods gave similar results for apoptosis). In some experiments, the cells were pretreated with 10 mM NH_4_Cl for 5 min (to raise the lysosomal pH, which largely prevented the uptake of the lysosomotropic compound AZM) and then exposed to AZM in the continued presence of NH_4_Cl (for details see the Methods section). In the non-oxidatively stressed cultures, cell death was < 3%. Values are the means ± 1 SD (n = 4; independently performed). Significant differences are indicated as follows: ** p < 0.01 and *** p < 0.001 (*vs.* controls) and ^§^ p < 0.05 (AZM + NH_4_Cl treated cells *vs.* AZM treated cells).

We also observed that AZM, H-DFO and NH_4_Cl caused an effective prevention of oxidant-induced cell death (Figure 4). To test whether or not the protection of J774 macrophages by AZM was due to the accumulation of this drug inside the lysosomes in a pH-dependent way, cultures were pretreated with NH_4_Cl before being exposed to AZM in the continued presence of NH_4_Cl. The normal pH inside the macrophage lysosomes is about 4.5, but upon addition of 10 mM NH_4_Cl, the lysosomal pH increases almost instantly. Under such circumstances, the cytoprotective effect by AZM was abolished (Figure 4).

Finally, we employed J774 macrophages to test whether or not AZM, H-DFO or NH_4_Cl had an influence on the cell features that are important for lysosomal Fe-reactivity, which are mainly Fe and the GSH and H-ferritin contents of the cells. However, such an interference of significance was thoroughly ruled out, and the results are summarized in Table 3.

**Table 3:** **The influence of different 4-hr treatments (for details see Methods section) on cell features that are decisive for lysosomal Fe**^**2+**^**-reactivity in murine 774 macrophages**

***ASSAYS***	**Cont.**	**AZM (μM)**		**H-DFO (mM)**	**NH**_**4**_**Cl (mM)**	**FeCl**_**3**_**(μM)**
		**33**	**660**	**2**	**10**	**100**
**Golde Index (n = 3)**	0.7 ± 0.8	0.6 ± 0.7	0.5 ± 0.5	0.2 ± 0.4	0.4 ± 0.6	194.3 ± 6.1***
**Cellular iron (ng/mg protein) (n = 4)**	0.2 ± 0.1	0.2 ± 0.0	0.2 ± 0.0	0.2 ± 0.0	0.2 ± 0.0	16.2 ± 2.3***
**H-ferritin (ng/mg protein) (n = 3)**	6.9 ± 1.4	8.2 ± 2.4	8.6 ± 1.7	7.6 ± 1.9	6.0 ± 1.8	109.8 ± 9.7**
**GSH (ng/mg protein) (n = 4)**	21.3 ± 2.0	20.7 ± 1.6	23.2 ± 3.3	20.2 ± 2.0	26.3 ± 3.9	26.3 ± 3.8*

To evaluate the different assays, the analysis was also performed on J774 macrophages that were exposed to 100 μM FeCl_3_ for 16 h. Data are presented as the means ± 1 SD. *, ** and *** indicate a significant difference from untreated control cells of p < 0.05, p < 0.01 and p < 0.001, respectively.

## Discussion

To the best of our knowledge, the present study is the first to demonstrate that the lysosomes of lung macrophages from lung transplant recipients are more susceptible to oxidative stress than the lysosomes of lung macrophages originating from healthy humans. Oxidant challenge led to a concomitant and pronounced lung allograft macrophage deaths (Figure 1). In contrast, macrophages from transplant recipients with AZM survived oxidant challenge significantly better and their lysosomes were efficiently protected against oxidants (Figure 1). This was despite the fact that macrophages from transplant recipients with AZM contained significantly more Fe and less GSH (Table 2), which both favor a pro-oxidative state of these macrophages. This observation does, indeed, indicate a significant protection afforded by AZM on oxidatively stressed lysosomes and cells. The differences noticed regarding Fe and GSH between the two groups of lung transplant macrophages is probably explained by macrophages with AZM being retrieved from much older transplants. Rejection and infection, other reasonable causes behind a sensitization towards oxidants, were not detected in any of the two groups.

Lung macrophages may provide an efficient defense against Fe-catalyzed oxidative lung tissue damage by harboring potentially harmful Fe inside their lysosomes [1-5]. Lung macrophages digest both organic Fe-containing elements, such as hemoglobin derived from red blood cells, and inorganic Fe-transporting particles, *e.g.,* silica [10-12]. Thus, in studies of lung macrophages that were harvested from a patient with ongoing pulmonary bleeding, we observed a dramatic increase of H-ferritin, which efficiently protected lysosomes and cells that were excessively loaded with Fe against oxidative injury [12]. H-ferritin is known to rapidly and copiously sequester reactive Fe [35], thereby acting as a custodian against Fenton-type chemistry outside as well as inside the lysosomes [4-6,10-13]. The latter is possible because the ferritin molecule normally is never completely Fe-saturated and therefore temporarily binds the lysosomal Fe in an un-reactive state before being degraded [4-6,12,13]. GSH contributes to the protection by scavenging free radicals in the cytosol and by working as an electron donor for glutathione peroxidases through which H_2_O_2_ is reduced to water [4,5,10-13].

However, this defense system by the lung macrophages might be over-whelmed if the cells and lysosomes are exposed to excessive Fe [10,11]. In a case of pulmonary alveolar proteinosis, we have previously shown that extensive amounts of reactive lysosomal Fe made lysosomes prone to rupture upon an oxidant-challenge that resulted in pronounced lung macrophage death [11]. This scenario might also explain the response to oxidant challenge observed in the present study in cultures of macrophages from lung transplant recipients without AZM (Figure 1). In vivo, extensive necrotic death of oxidant-exposed lung macrophages results in the release not only of harmful hydrolytic enzymes to the epithelial lining fluid (ELF) from the interior of disrupted lysosomes [36] but also of reactive Fe from the same organelle [2-5]. This is probably a major reason behind the increased amounts of reactive ELF-Fe, which subjects the respiratory epithelium to increased oxidative stress [4,5], that is also observed in many inflammatory lung diseases [2,3].

Analyses of the possible reasons behind the observed susceptibility of lysosomes and cells to an oxidant challenge reveal that the anti-oxidant status of macrophages from transplant recipients without AZM is changed toward a pro-oxidative state. This is probably the result of significantly increased amounts of Fe, which are not sufficiently compensated for by an increase of H-ferritin (Table 2). The amounts of GSH were similar in the macrophages from both the transplant recipient without AZM and the healthy subjects (Table 2). Although we emphasize the increased lysosomal Fe reactivity as the most likely reason for a significantly increased susceptibility of lysosomes in lung transplant macrophages without AZM to oxidative stress, we also notice a significant gender mismatch in the present study (Table 1). A gender dependency for the lysosomal susceptibility to oxidative stress seems unlikely but cannot be ruled out by our current research on lung macrophages.

All lung transplant recipients, without AZM or with AZM, were on prophylaxis treatment with omeprazol against gastro-esophageal reflux. Since this drug is a proton pump inhibitor, theoretically, it has the potential to inhibit AZM-mediated cytoprotection by blocking the lysosomal uptake and accumulation of AZM through alkalinizing the lysosomes [37,38]. However, lysosomes of macrophages from transplant recipients with AZM were efficiently stabilized in contrast to the lysosomes of the macrophages without AZM. This finding strongly speaks against this theory and is, in fact, well in line with previous studies of different cell types demonstrating no effect of omeprazol on lysosomes regarding their integrity [39,40], activity of lysosomal enzymes [39,40] or their acidification [41].

AZM is known to cause an accumulation of phospholipids and cholesterols inside lysosomes, which is an effect mediated by the interaction with phospholipids and a blockage of phospholipase A1 [42,43]. This lysosomal accumulation (*i.e.,* lysosomal phospholipidosis) has previously been proven to greatly increase the lysosomal resistance to oxidative stress [44,45]. All transplant recipients with AZM were on this treatment since at least a month before lung macrophages were harvested and studied. Clearly, this long-term effect by AZM on lysosomes of macrophages from transplant recipients treated with the same drug cannot be ruled out as a significant mechanism behind the protection observed in the present study.

Similar to NH_4_Cl, AZM may also decrease the lysosomal pool of Fe by raising the pH inside the acidic vacuome (late endosomes and lysosomes), thus blocking the uptake of Fe from the transferrin-transferrin-receptor complex occurring in the late endosomes [46] and/or preventing the enzymatic liberation of Fe from Fe-containing organic material accumulating inside the lysosomes [24,26,29,47]. However, assessment of Prussian-blue stained Fe in the macrophages (*i.e.*, the Golde index) from transplant recipients with AZM did not indicate that this scenario occurs in vivo. In contrast, the Golde index of macrophages from recipients with AZM tended to be higher, not less, than that of macrophages from recipients without AZM. Moreover, protection of J774 macrophages against oxidative stress was afforded by only a 45-min AZM exposure. It takes many hrs of lysosomal alkalinization to achieve this effect on the lysosomal pool of Fe and its reactivity [29,47]. Moreover, a short exposure to AZM (4 hrs) had no significant effect on the cellular content of Fe, GSH and H-ferritin in J774 macrophages. Collectively, these findings do, indeed, suggest a more immediate mode of action by AZM on lysosomes. Importantly, test tube experiments indicate that AZM forms a relatively stable 1:1 complex with Fe^2+^[48]. The lactone ring in the AZM molecule is substituted with a number of hydroxyl and amine functional groups that are positioned in a suitable configuration for interaction with Fe.

H-DFO is a potent chelator of Fe, which exclusively targets the lysosomal pool of Fe [30]. This is because H-DFO is a high-molecular-weight neutral polymer that cannot be degraded by the lysosomal enzymes, and, thus, remains permanently inside the lysosomal compartment after endocytic uptake [30]. AZM and H-DFO prevented LMP and cell death to a similar extent (Figure 3 and Figure 4). On a molar basis, AZM was 60 times more efficient than H-DFO. AZM rapidly accumulates inside the cells and lysosomes through a concentration- and pH-dependent passive diffusion [24-26], while the fluid-phase endocytosis of H-DFO is far less effective and a slower process for a lysosomal deposition of drugs. Thus, AZM is lysosomotropic, which has been demonstrated in previous studies on a number of cell types including J774 macrophages [24-26]. In line with these previous findings we demonstrated an almost complete loss of cell protection by AZM in the presence of a brief exposure to NH_4_Cl that raised the pH of the lysosomes (Figure 4).

Overall, our findings are consistent with previous research demonstrating a disturbed Fe metabolism in lung transplants [49-51]. However, the present study expands our understanding greatly about how oxidative damage in the lung transplant may be mediated, pointing out the important role of lysosomes. The observations of the present study also indicate that AZM effectively prevents oxidative damage on lysosomes and concomitant cell death. It seems that AZM exerts an immediate effect on a currently unknown lysosomal product that is harmful during an oxidant challenge. We believe that the most likely candidate would be free or loosely bound lysosomal Fe that is in a reactive state, but further studies are warranted.

## Conclusions

The present study is the first to show that lung macrophages derived from human lung transplant recipients, as compared to lung macrophages of healthy subjects, are sensitized to oxidative stress in cultures and have lysosomes that are more prone to leakage due to a disturbed balance of Fe. Moreover, lung macrophages (and their lysosomes) become significantly less susceptible to oxidant challenge in vitro, when treated with the lysosomotropic antibiotic AZM in vivo. Collectively, experiments on transplant lung macrophages and murine J774 macrophages suggest that protection against oxidants afforded by AZM is mediated by this drug attenuating the reactivity of the lysosomal pool of Fe. Given that oxidant-induced LMP and lung macrophage death is of some importance for BOS development in lung transplants, we propose that the preventive effect of AZM against BOS is at least partly due to its protective effects on lysosomes and cells.

## Abbreviations

AO: Acridine orange; AZM: Azithromycin; BAL(F): Bronchoalveolar lavage (fluid); BOS: Bronchiolitis obliterans syndrome; DMEM: Dulbecco's modified eagle's medium; ELF: Epithelial lining fluid; GO: Glucose oxidase; GSH: Reduced glutathione; H-DFO: High-molecular-weight desferrioxamine; LMP: Lysosomal membrane permeabilization; MSDH: *O*-methyl-serine dodecylamide hydrochloride; OB: Obliterative bronchiolitis; PBS: Phosphate-buffered saline solution; ROS: Reactive oxygen species.

## Competing interests

None of the authors have any conflicts of interest, financial or non-financial, to disclose.

## Authors' contributions

HLP designed the study, performed bronchoscopies, wrote the manuscript and obtained grant fundings. LKV performed experiments and wrote parts of the manuscript. MS, JP, SDL and UW contributed to the manuscript writing and MS also performed bronchoscopies. All authors have given their final approval of the version submitted. This study was performed at the Divisions of Pulmonary Medicine and Experimental Pathology, Linköping University, Sweden

## Financial support

This work was supported by grants to HLP from the County Council of Östergötland (ALF), Sweden, the Medical Research Council of Southeast Sweden (FORSS), the Swedish Medical Society and Linköping Medical Society, and the Research Funds of Olle Engkvist, Apotekare Hedberg and LiÖ (Östergötland, Sweden).
